# Microplastic Quantification in Aquatic Birds: Biomonitoring the Environmental Health of the Panjkora River Freshwater Ecosystem in Pakistan

**DOI:** 10.3390/toxics11120972

**Published:** 2023-11-30

**Authors:** Muhammad Bilal, Atif Yaqub, Habib Ul Hassan, Sohail Akhtar, Naseem Rafiq, Muhammad Ishaq Ali Shah, Ibrar Hussain, Muhammad Salman Khan, Asad Nawaz, Salim Manoharadas, Mohammad Rizwan Khan, Takaomi Arai, Patricio De Los Ríos-Escalante

**Affiliations:** 1 Department of Zoology, Government College University Lahore, Lahore 54000, Pakistan; m.bilaldigaan@gmail.com (M.B.); atif@gcu.edu.pk (A.Y.); 2 Department of Zoology, University of Karachi, Karachi 75270, Pakistan; 3 Fisheries Development Board, Ministry of National Food Security and Research, Islamabad 44000, Pakistan; 4 Department of Mathematics and Statistics, University of Haripur, Haripur 22620, Pakistan; s.akhtar@uoh.edu.pk; 5 Department of Zoology, Abdul Wali Khan University, Mardan 23200, Pakistan; naseem@awkum.edu.pk (N.R.); salman.khan@awkum.edu.pk (M.S.K.); 6 Department of Chemistry, Abdul Wali Khan University, Mardan 23200, Pakistan; 7 Department of Statistics, Government College University Lahore, Lahore 54000, Pakistan; 8 Institute for Advanced Study, Shenzhen University, Shenzhen 518060, China; 007298@yzu.edu.cn; 9 Department of Botany and Microbiology, College of Science, King Saud University, Riyadh 11451, Saudi Arabia; smanoharadas@ksu.edu.sa; 10 Department of Chemistry, College of Science, King Saud University, Riyadh 11451, Saudi Arabia; mrkhan@ksu.edu.sa; 11 Environmental and Life Sciences Programme, Faculty of Science, Universiti Brunei Darussalam, Gadong BE 1410, Brunei; takaomi.arai@ubd.edu.bn; 12 Facultad de Recursos Naturales, Departamento de Ciencias Biológicas y Químicas, Universidad Católica de Temuco, Temuco 4780000, Chile; prios@uct.cl

**Keywords:** microplastic pollution, freshwater, aquatic birds, Panjkora River, Pakistan

## Abstract

Microplastic pollution has become a global concern, with potential negative impacts on various ecosystems and wildlife species. Among these species, ducks (*Anas platyrhynchos*) are particularly vulnerable due to their feeding habits and proximity to aquatic environments contaminated with microplastics. The current study was designed to monitor microplastic (MP) pollutants in the freshwater ecosystem of the Panjkora River, Lower Dir, Pakistan. A total of twenty (20) duck samples were brought up for four months and 13 days on the banks of the river, with no food intake outside the river. When they reached an average weight of 2.41 ± 0.53 kg, all samples were sacrificed, dissected, and transported in an ice box to the laboratory for further analysis. After sample preparation, such as digestion with 10% potassium hydroxide (KOH), density separation, filtration, and identification, the MP content was counted. A total of 2033 MP particles were recovered from 20 ducks with a mean value of 44.6 ± 15.8 MPs/crop and 57.05 ± 18.7 MPs/gizzard. MPs detected in surface water were 31.2 ± 15.5 MPs/L. The major shape types of MPs recovered were fragments in crop (67%) and gizzard (58%) samples and fibers in surface water (56%). Other types of particles recovered were fibers, sheets, and foams. The majority of these detected MP particles were in the size range of 300–500 µm (63%) in crops, and 50–150 µm (55%) in gizzards, while in water samples the most detected particles were in the range of 150–300 µm (61%). Chemical characterization by FTIR found six types of polymers. Low-density polyethylene (LDPE) had the greatest polymer detection rate (39.2%), followed by polyvinyl chloride (PVC) (28.3%), high-density polyethylene (HDPE) (22.7%), polystyrene (6.6%), co-polymerized polypropylene (2.5%), and polypropylene homopolymer (0.7%). This study investigated the presence of microplastics in the crops and gizzards of ducks, as well as in river surface water. The results revealed the significant and pervasive occurrence of microplastics in both the avian digestive systems and the surrounding water environment. These findings highlight the potential threat of microplastic pollution to wildlife and ecosystems, emphasizing the need for further research and effective mitigation strategies to address this pressing environmental concern.

## 1. Introduction

Over the past few decades, the ubiquity and persistence of microplastic pollution have become a growing concern worldwide [1,2]. Global plastic production has increased the threat to society by contaminating the environment [3]. Particularly in aquatic environments, microplastics are contributing to excessive water pollution, and the ingestion of these particles is suspected to be harmful to organisms [1,3]. Microplastics, defined as particles smaller than 5 mm in size, have emerged as a significant environmental threat due to their abundance and potential to accumulate in various ecosystems [4,5]. Microplastics can enter freshwater environments in several ways, mostly via float-up garbage and waste material, but also through shared drain overflows, and degraded plastic debris from industrial effluent [6]. The issue of microplastic pollution gets worse as plastic particles break down into smaller particles as a result of various physical, chemical, and environmental factors [2,3,4]. Based on the sources, microplastics are divided into primary and secondary categories [3,7]. Small pieces of plastic called primary microplastics can enter the environment directly or indirectly through an overspill. Spills, sewage, and domestic and industrial effluent are examples of direct emissions of primary microplastics [8]. Secondary microplastics are formed from larger plastic items present in the environment when these larger plastic items disintegrate into smaller particles due to different physical, chemical, and environmental factors [6,9]. The amount of microplastic pollution is growing every day, increasing the risk of ecosystem exposure, and it is estimated that eight million tons of polyethylene bags leak into our aquatic ecosystem every year [6]. If plastics are produced and managed at the current rate, 12 billion tons of plastic waste will be discarded in landfills or the natural environment by 2050 [10]. Once in the environment, abiotic and biotic processes involving chemical, physical, and biological reactions can lead to the degradation of plastic waste at a very slow rate, generating numerous smaller plastics—and plastic manufacturing is expected to double [10]. Because of this increased risk of particle interaction with organisms, ingestion, adsorption, physical entanglement, and dangerous impacts across food webs, microplastics have the potential to have a wide range of effects on biota [3,11,12].

Plastic waste and debris have caused substantial environmental pollution globally in recent decades, and they have accumulated in hundreds of terrestrial and aquatic avian species. Birds are susceptible and vulnerable to external environments; therefore, they could be used to estimate the negative effects of environmental pollution [6,11]. Aquatic birds are especially susceptible to the negative impacts of microplastics because of their dependence on marine and freshwater ecosystems [6,13]. They perform important ecological roles as markers of environmental health and biodiversity since they are a diversified collection of avian species that live in coastal and inland environments [14]. The uptake of microplastics by aquatic birds is a sign of the intricate relationships that exist between avian species and their surroundings. These minute synthetic polymer pieces, which come from plastic bottles, fishing nets, and microbeads, are small enough to be mistaken for food [15]. Birds unintentionally swallow these particles with their prey or directly from contaminated water as they hunt in water bodies teeming with microplastics. As a result, microplastics may build up inside the bodies of the birds, which may have a variety of negative physiological, behavioral, and ecological effects [3,13,14].

Numerous studies have shown that microplastics (MPs) are ingested by aquatic, terrestrial, and avian species and retained in different sections of the gastrointestinal tract [10,16,17]. Ingestion and retention can be linked to a variety of physical, physiological, neural, and hormonal issues, chiefly a reduction in the area of the intestine that is used to absorb nutrients, inhibition of gastric and pancreatic enzymatic activity, decreased steroid hormones, delayed ovulation, cytotoxicity, lipid oxidative damage in gills and muscle, and neurotoxicity through lipid oxidative damage [3,5,10,11,13]. Microplastics can act as carriers for toxic chemicals, and contain many organic and inorganic contaminants, including heavy metal polychlorinated biphenyl (PCB) pollutants, the hazardous impacts of which are important to consider because these MPs further increase the likelihood of toxicity [5,7,11,18]. MPs absorb and excrete heavy metals within living organisms, for instance in the digestive tract, where desorption is facilitated by a low-pH environment [3]. The ingestion of microplastics by aquatic birds is a prevalent concern due to their feeding habits and the high abundance of microplastics in their foraging environments [14]. In wildlife, there are increasing reports of the ingestion of plastics across a wide range of taxa, including birds, with detrimental effects on health [7,10]. These effects include physical impairments such as intestinal blockage, ulcers, and perforation of the gut, and fake satiety as well as toxicological effects such as reproductive disorders, activation of inflammatory responses, and immunodeficiency, which might lead to increased mortality [4,10]. A study by Neves [19] found that 50% of ingested prey items in northern gannets (*Morus bassanus*) from the North Sea contained microplastics. Similarly, a study by Provencher [20] reported the presence of microplastics in the gastrointestinal tracts of multiple species of seabirds, including northern fulmars (*Fulmarus glacialis*) and black-legged kittiwakes (*Rissa tridactyla*). Microplastics can have detrimental physiological effects on aquatic birds, primarily through mechanical obstruction, inflammation, and chemical toxicity. A study by Ziccardi [21] demonstrated that ingestion of microplastics resulted in gut obstruction, reduced feeding efficiency, and altered nutrient absorption in common murres (*Uria aalge*). Additionally, Rummel [22] found that microplastics induce oxidative stress, inflammation, and tissue damage in several seabird species, including great shearwaters (*Ardenna gravis*) and Cory’s shearwaters (*Calonectris diomedea*). A study by Bour [23] observed that microplastics in greater scaup (*Aythya marila*) contained higher concentrations of persistent organic pollutants compared to the surrounding sediments, indicating their potential to transfer chemicals to higher trophic levels. The ingestion of microplastics has been linked to immunotoxic effects in aquatic birds. Bond [24] found that exposure to microplastics resulted in suppressed immune responses in European herring gulls (*Larus argentatus*). Additionally, it caused endocrine disruption, fertility impairments, and reduced reproductive success in European starlings (*Sturnus vulgaris*).

Recently, studies have been published across the globe to report and highlight the MPs issue in birds, especially in aquatic ecosystems [6,11,13,14,25,26,27]. However, there has been no such attempt from Pakistan to highlight this concerning issue. Understanding the severity of the impact of microplastic pollution on aquatic birds necessitates a comprehensive assessment of their ecological significance. The present study aims to contribute to the growing body of knowledge regarding microplastic pollution in aquatic birds. By addressing the sources, effects, and implications of microplastics on avian health and their ecosystems, we hope to shed light on this emerging environmental issue and stimulate further research towards effective conservation strategies.

## 2. Material and Methods

### 2.1. Duckling Rearing and Sacrifice

**Figure 1:**
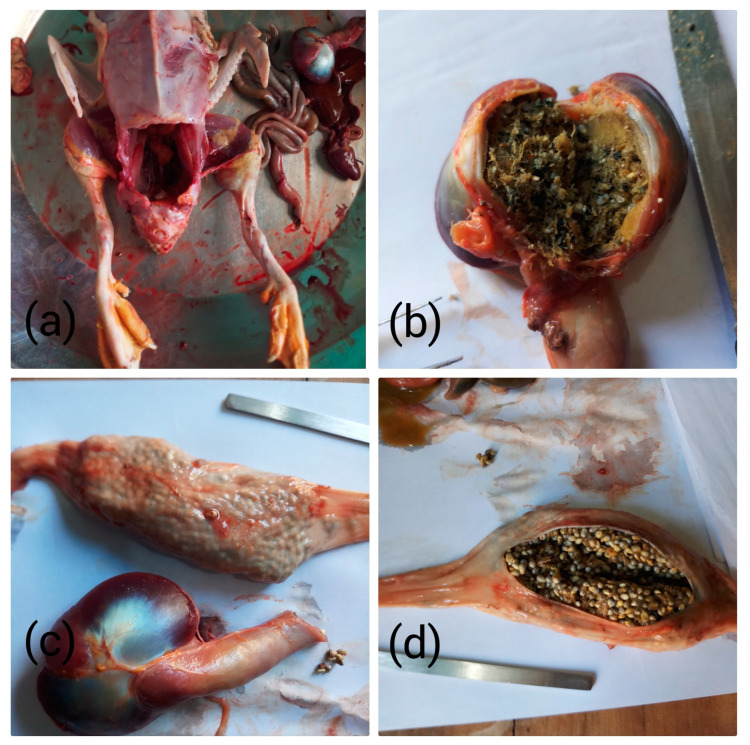
Dissection of duck: (**a**) Open gizzard with semi-digested content. (**b**) Closed crop and gizzard. (**c**) Open crop with ingested content (**d**).

A total of twenty (20) duckling (*Anas platyrhynchos*) samples were hatched and brought up for four months and 13 days on the banks of Panjkora River (34.768449, 71.792282), Lower Dir, KP, Pakistan. The total food intake of the ducklings was from the river. When they reached an average weight of 2.41 ± 0.53 kg, all samples were sacrificed and dissected, having had no food intake outside the river, and the crops and gizzards were transferred to beakers, labeled, and stored in the freezer for further analysis (Figure 1). Surface water samples were collected with a 1 L glass jar. The lid was removed, and then the glass jar was dipped into the water and samples were taken just one inch below the surface. When the jar was filled, it was recapped and stored in an ice box [7].

#### Sample Preparation (Digestion, Density Separation, and Filtration)

Potassium hydroxide 10% (KOH) was added to a beaker containing crop and gizzard at a ratio of 5:1 to digest the sample. Then, it was stored in a water bath for 36 h at 55 °C. KOH was suggested for digestion since it is believed to have little effect on the decomposition of microplastics during the digestive process [3]. Density separation was used to separate the microplastic components. Sodium chloride (3:1 *v*/*v*) was added after digestion, and the mixture was agitated for 20 min before settling for 24 h [12]. After a 24 h period of settlement, the sample’s supernatant layer was removed, and three distinct sizes of fractions were obtained by passing each through sieves with varying pore sizes (500 µm, 300 µm, 150 µm, and 50 µm). Each fraction was then filtered using filter paper in a filtering assembly. After filtration, the filter assembly cup’s walls were cleaned twice, and the solid-containing filter paper was left in a Petri dish to dry for a day before being ready for detection [3,12]. The same procedure was followed for water samples except that in the digestion step, Fenton’s reagent, an acidic solution of ferrous sulfate, and hydrogen peroxide (35%) were added to 200 mL of the sample, respectively, to digest any organic compounds that might have been present.

### 2.2. Microplastics Observation, Identification, and Quantification

A light binocular microscope (at 16 × 4 and 16 × 10 magnification, Labomed, model: CXL-110446002, 9135002, New York, NY, USA) was used for observation and inspections of dried filter papers containing particles. All particles of MPs were counted manually under microscopic observation. Images of the discovered particles were taken using a 1600× USB 8 LEDs electronic digital microscope camera and a Zeiss stereomicroscope Stemi 508 microscope at 2.5 magnification. In the current study, we did not measure any particles smaller than 50 µm. To identify categories, physical attributes including size, geometry, and color were taken into account. The polymer spectrum library of Omnic Spectra (Version 7.3, Thermo Fisher Scientific Inc., Waltham, MA, USA) software was used to perform polymer identification FTIR spectroscopy (IRTracer-100, Shimadzu, Columbia, MD, USA).

### 2.3. Background Contamination Control and Limitations of The Study

To keep samples from being contaminated by air, every safety precaution was taken. When not in use, all laboratory tools, including glassware and chemicals, were covered with aluminum foil. To prevent environmental contamination, distilled water, reagents, and other materials were filtered and covered in aluminum foil. To determine the suspended load of MPs from the environment, a few filter papers were scattered throughout the lab for 72 h in various locations. These filter sheets were then viewed with a stereomicroscope. Six of these filter sheets were evaluated and preserved as a control for the analysis. Any particle less than 50 µm was not considered in this study due to the non-availability of equipment for fine detection.

## 3. Results and Discussion

**Figure 2:**
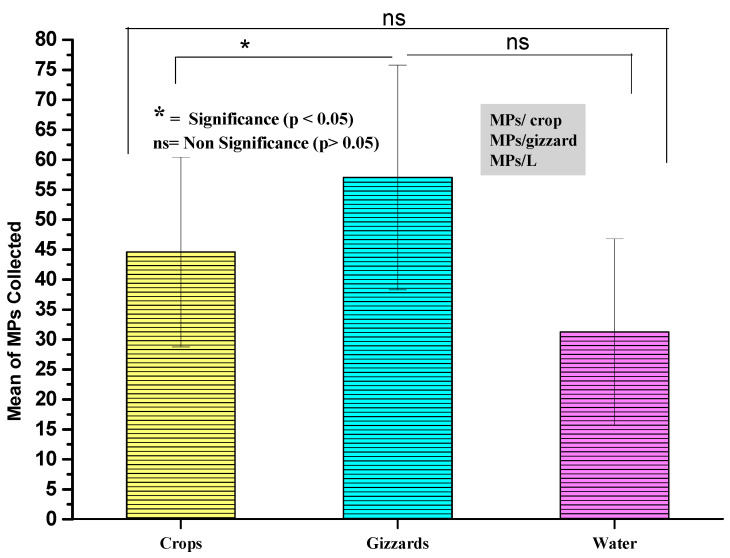
The mean concentration of MPs detected in crops, gizzards, and river water.

**Figure 3:**
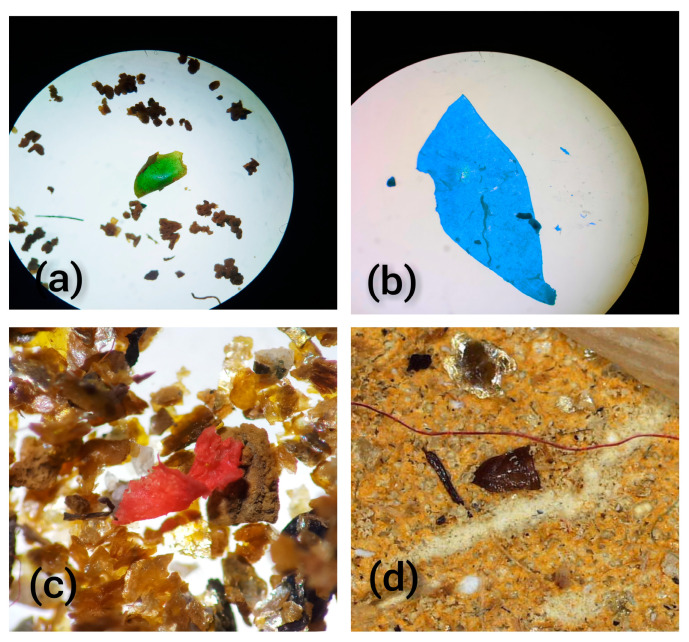
Microscopic images of MP fragment (**a**), sheet (**b**), foam (**c**), and fiber (**d**).

A total of 2033 MP particles were recovered from 20 duck samples combined from crops and gizzards, where 892 MP particles were recovered from 20 crop samples of ducks with a mean value of 44.6 ± 15.8 MPs/crop, and 1141 MPs from gizzard samples with a mean value of 57.05 ± 18.7 MPs/gizzard. Meanwhile, a total of 625 MP particles were detected from 20 samples of surface water of the river in which these ducks were reared. The mean of MPs detected in surface water was 31.2 ± 15.5 MPs/L (Figure 2 and Figure 3). A weak correlation was seen to exist between the concentration of MPs in river water and the crop of a duck (r = 0.24), while a very weak correlation was observed between the concentration of MPs in river water and gizzards (r = 0.058) (Table 1). The possible potential reason for the weak correlation may be the loss of MP particles from crops and gizzards through the gastrointestinal canal in feces. Various studies across the globe have highlighted the MPs issue in aquatic birds; for example, Susanti et al. [28] collected 25 duck samples for the assessment of MPs. They found 27 to 41 MPs/individual in the gastrointestinal tracts of the ducks. Another study, by Bustamante [29], assessed and found MPs in the gizzards of Virginia waterfowl. In his study, the author assessed some species such as mallard (Anas platyrhynchos), long-tailed duck (Clangula hyemalis), goldeneye duck (Bucephala clangula), Canada goose (Branta canadensis), and ringneck duck (Aythya collaris). He recovered MP particles from 53.6% of the gizzards of waterfowls. The abundance range he found was 0 to 1.75 MPs/gram of gizzard material. Faure et al. [30] evaluated and carried out sampling in Lake Geneva, gathering samples from the following species: *Cygnus olor* (Gmelin, 1789), *Anas platyrhynchos* (L., 1758), and Ardea cinerea (Linnaeus, 1758); MPs were found in the gastrointestinal (GI) tracts of eight of the nine birds. When further *Cinclus cinclus* (Linnaeus, 1758) specimens were taken, regurgitates and fecal samples were used to determine the prevalence of MPs, which was discovered to be 50% and 45%, respectively [31]. A broader study investigated the MP ingestion in 350 samples from 17 species, including a marine one. It revealed an anthropogenic debris ingestion rate of 11.1%. According to an extrapolation of the findings limited to plastic, 9.7% of freshwater species include MPs [32]. In addition to research on adult birds, Laurentian Great Lakes’ *Phalacrocorax auritus* (Lesson, 1831) chicks were dissected. According to Brookson et al. [33], the majority of MP fibers were found in the gastrointestinal (GI) tracts of over 86% of the chicks. Numerous studies have examined how marine debris is consumed by seabirds [34] and microplastics, which are essentially pellets and user fragments, have been isolated from cadavers, regurgitated samples, and feces of birds used in the studies [35,36,37,38]. Seabirds may be able to regurgitate microplastics from their digestive tracts after consumption [39]. On the other hand, this implies that parents might expose their chicks to plastic while feeding them. This is corroborated by Kühn and van Franeker’s [40] discovery that juvenile intestines contain more plastic than adult ones. This may suggest that the majority of bird microplastic contamination occurs between generations and that the act of regurgitation may cause the degradation of microplastics into even smaller particles. The majority of the birds investigated did not pass away as a direct result of ingesting microplastic, hence it may be deduced that seabirds are not as seriously harmed by microplastic ingestion as they are by macroplastic ingestion [41]. There is currently no evidence that microplastics can cross the intestine barrier, enter the bloodstream, or accumulate in various organs because the majority of studies on microplastics in seabirds only examined microplastics in the digestive tract and feces [42]. No research has shown nanometer-sized microplastics in the excrement or intestines of seabirds as of yet. (Table 2).

### 3.1. Shapes of Detected MPs

**Figure 4:**
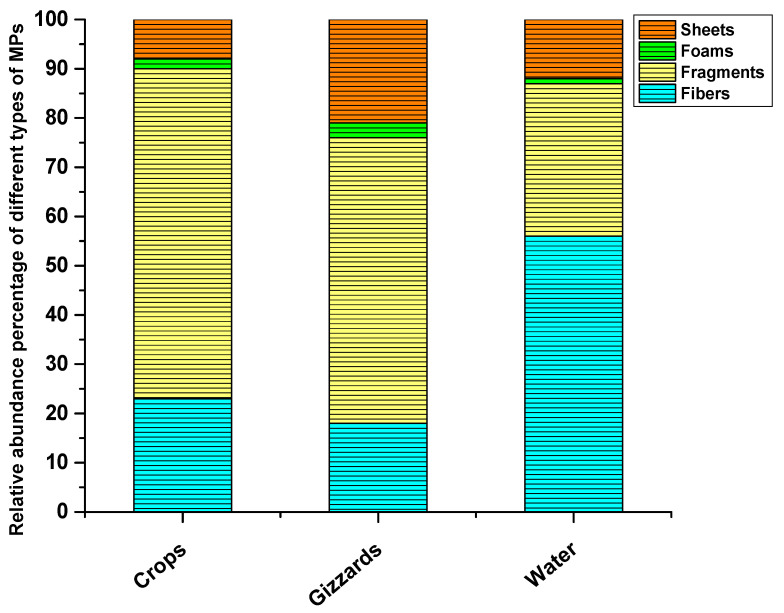
Different shape types of MPs detected.

The most common shape types of MPs recovered were fragments in crop and gizzard samples and fibers in surface water. Of the total MPs detected in crops, 67% were fragments, while the other types of particles recovered from crops were fibers, sheets, and foams, with percentages of 23%, 8%, and 2%, respectively. Fragments were also the dominant type of MPs in gizzards as 58% of the detected MPs were fragments in gizzards. Other types of MPs found in gizzard samples were sheets (21%), fibers (18%), and foams (3%). Surprisingly, the dominant types of MPs detected in water samples were fibers with an abundance percentage of 56%. Fragments were the second most abundant type of particle in water samples, at 31%, while sheets and foams were 12% and 1%, respectively (Figure 3 and Figure 4, Table 2). The majority of microplastic particles detected in avian gizzards and crops are fragments. For example, Collard [43] observed that 72.9% of the particles were fragments. Tokunaga [44] identified fragments as the predominant kind of microplastics in their investigation of wild birds in Japan. Zhao [45] found that fragments were a crucial sort of particle shape for the retrieved microplastics. In their research, 54.9% of MPs were fragments, while 37.4% were fibers. Unlike the current study, Deoniziak [46] indicated that fragments made up only 10% of the total particles and that fibers were predominant (84%) among the particles. Less mobility in the GIT tract and difficulty excreting through feces may be contributing factors to the preponderance of fragment-type particles in crops and gizzards. Another scenario is that the plastic pieces fool the birds into thinking they are food, and they eat them selectively (Table 2).

### 3.2. Sizes of Detected MPs

**Figure 5:**
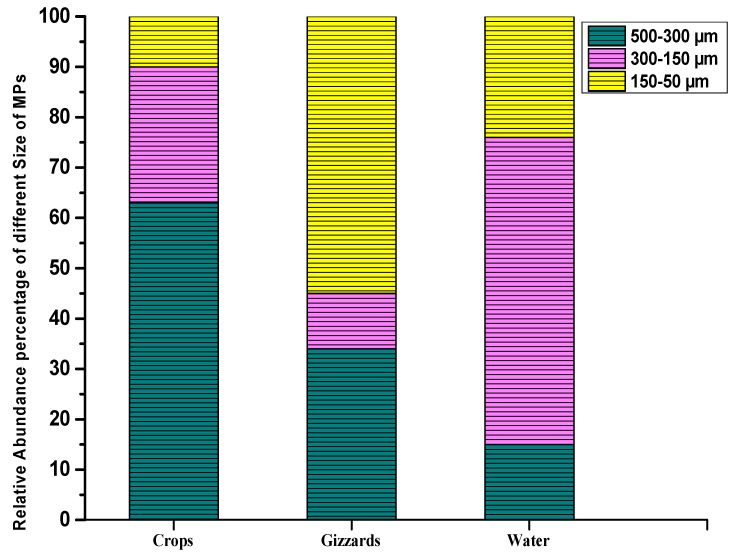
Size ranges of the detected MPs.

In terms of size range, 63% of these detected MP particles were in the size range of 500–300 µm in crops, while the abundances of other size ranges of detected particles in crops were 27% (150–300 µm) and 10% (50–150 µm). Unlike in crops, the dominant size range of detected MPs in gizzards was 150–50 µm, at 55%. The abundance percentages of other size ranges in gizzards were 34% (300–500 µm) and 11% (150–300 µm). Meanwhile, in water samples, the most detected particles were in the range of 150–300 µm (61%). Other size ranges such as 300–500 µm and 50–150 µm were 15% and 24%, respectively (Figure 5 and Table 2). Various investigations conducted around the world have shown high concentrations of comparably bigger particles. Bessa et al. [47] retrieved 19 microplastic particles from the scat of penguins where the majority of the discovered particles were greater than 500 µm, and the mean size of the particles was 1266 ± 1378 µm. Liu [48] reported the presence of microplastics, with the majority of the particles being in the 500–1000 µm size range. Particles from the gastrointestinal (GI) tracts of birds were collected by Zhu [49] and included 92.9% of particles smaller than 5 mm, and more than 90% in a study by Zhao [45], while Deoniziak [46] gathered bird GIT particles that were smaller than 1000 µm. The majority (68.7%) of little-black cormorants’ (Phalacrocorax sulcirostris) GIT-extracted particles were in the 100–1000 µm size range [50]. The fact that larger particles are less mobile in the GIT tract and consequently get stuck in different regions of the GIT, such as the stomach and gizzards, may be the cause of the significantly larger particle presence. The smaller particles, however, tend to pass through feces and are more easily moved by the GIT (Table 2).

### 3.3. Detected Polymer Types of MPs

**Figure 6:**
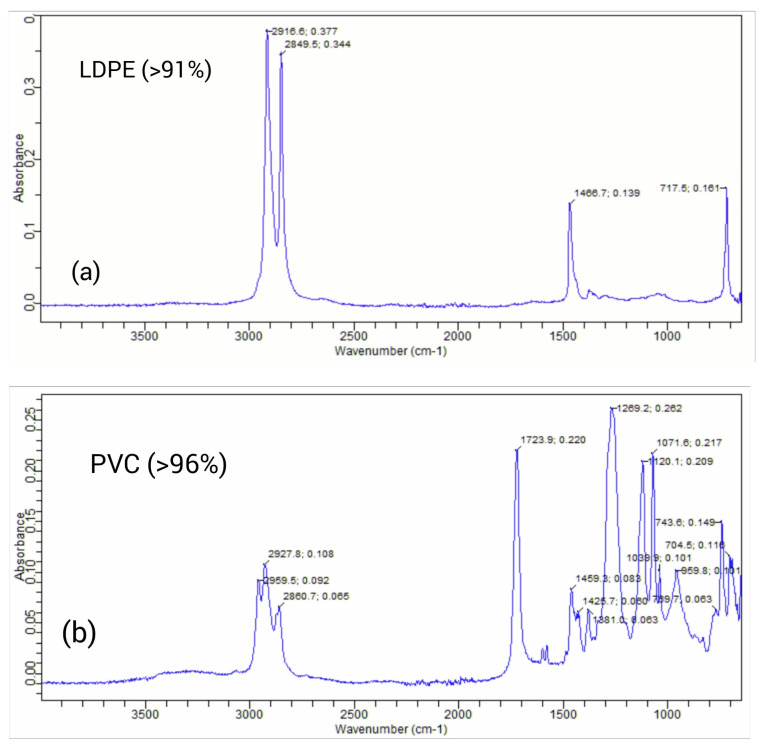
The top two most abundant types of polymers: LDPE (**a**) and PVC (**b**).

It was feasible to establish the chemical composition of the type of polymer by employing FTIR spectroscopy [51]. The absorbance peaks were noted after measuring the MP particles with an ATR sensor. The peak similarity index was additionally employed to evaluate the particle composition by contrasting recorded and reference peaks. Low-density polyethylene (LDPE), polyvinyl chloride (PVC), high-density polyethylene (HDPE), polystyrene (PS), co-polymer polypropylene (COPP), and polypropylene homopolymer (PPH) were the six types of polymers found. Low-density polyethylene (LDPE) had the greatest detection rate (39.2%), followed by polyvinyl chloride (PVC) (28.3%), high-density polyethylene (HDPE) (22.7%), polystyrene (6.6%), co-polymerized polypropylene (2.5%), and polypropylene homopolymer (0.7%) (Figure 6 and Table 2). While several studies have shown various polymer types, there was considerable global overlap in the polymer types found in microplastic particles. The majority of the microplastic particles, according to Collard [52], were of the polypropylene, polystyrene, and polyethylene variety. Polyethylene was identified as the most prevalent form of polymer. In a different study, polyethylene terephthalate (16%), ethylene-co-polypropylene (11%), and cellulose were shown to be more prevalent than the previous two types of polymers, making up 37% of the total particles [26]. In their research, Bessa et al. [47] discovered several polymers, including polypropylene, polyethylene, polyacrylonitrile, and polyacrylate. Other studies [51] have also referred to these polymers as being among the primary types of MPs. The majority of packaging employs LDPE and HDPE polymers, including foils, milk, shampoo, oil, and soap bottles, domestic items like trays, plates, and cups, cables, and PVC in electrical and electronic equipment, tour tents, and water pipes [9]. Packaging for a variety of items, such as structural tanks, battery covers, and pump components, has been made with PPH [53]. All of these plastic items, when thrown into or close to water bodies, shatter into small pieces and finally lead to the production of MPs with different polymer natures. In the current study, the major fraction of the load of these polymer types of MPs comes from wastewater discharge into the river from the local market containing factories of plastic pipes, spices, plastic shoes, etc., and also tourists frequently leaving behind disposable plates, glasses, water bottles, food wrappers, and other items near and in the river. It was concluded as a result that the identified polymers in this study were related to their potential application in the region under study (Table 2).

**Table 2:** Comparison of the present study with worldwide reports.

Particles	Region	Detected Particles	Reference
	Pakistan	MPs found with a mean of 44.6 ± 15.8 MPs/crop and 57.05 ± 18.7 MPs/gizzard of duck	Present Study
	Pakistan	Extracted 33.25 ± 17.8 MPs/gizzard, 17.8 ±12.1 MPs/crop of bird	[12]
	Indonesia	Found 27 to 41 MPs/duck	[28]
	Virginia, USA	Found 0 to 1.75 MPs/gram of gizzard of Virginia fowl	[29]
	Zurich and Brienz, Switzerland	MPs were present in eight of the nine birds	[30]
	South Wales, UK	MPs were found in 50% of regurgitates (n = 72) of Eurasian dipper (*Cinclus cinclus*)	[31]
	Canada	MPs found in 9.7% of freshwater species	[32]
	North America	MP fibers were found in the GI tracts of over 86% of the chicks of diving birds	[33]
	Norway	15–106 MPs from northern fulmar (*Fulmarus glacialis*)	[37]
	Antarctic regions (Bird Island, South Georgia and Signy Island, South Orkney Islands)	Retrieved 19 microplastic particles from the scat of penguins	[47]
Size	Region	Dominant size range detected	Reference
	Pakistan	The majority (63%) of the detected MP particles were in the size range of 500–300 µm in crops; the dominant (55%) size range of detected MPs in gizzard was 150–50 µm	Present Study
	Pakistan	300–500 µm	[12]
	China	More than 90% of particles were smaller than 5 mm	[45]
		Particles from bird GIT were smaller than 1000 µm	[46]
	Antarctic regions (Bird Island, South Georgia and Signy Island, South Orkney Islands)	The majority of the discovered particles from the scat of penguins were greater than 500 µm	[47]
	China	The majority of the particles were in the 500–1000 µm size range	[48]
	China	Of the total particles from the GIT of the birds collected, 92.9% were particles smaller than 5 mm	[49]
	Indonesia	The majority (68.7%) of the little-black cormorant’s (Phalacrocorax sulcirostris) GIT-extracted particles were in the 100–1000 µm size range	[50]
Shape	Region	Dominant shape type detected	Reference
	Pakistan	67% of total detected MPs from crops were fragments while 58% of detected MPs were fragments in gizzards	Present Study
	Pakistan	Fragments (64%) in gizzards Fragments (53%) in crops	[12]
		The majority (72.9%) of microplastic particles detected in avian gizzards and crops were fragments.	[43]
	Japan	Fragments as the predominant kind of microplastics detected in their investigation of wild birds in Japan	[44]
	China	In this study, 54.9% were fragments, while 37.4% were fibers.	[45]
	Poland	Fragments made up only 10% of the total particles and fiber was predominant (84%) among the particles in common blackbirds (*Turdus merula*) and song thrushes (*Turdus philomelos*).	[46]
Polymer	Region	Polymer types detected	Reference
		Low-density polyethylene (LDPE) had the greatest polymer detection rate (39.2%), followed by polyvinyl chloride (PVC) (28.3%), and high-density polyethylene (HDPE) (22.7%); 6.6% of the material was polystyrene, 2.5% was co-polymerized polypropylene, and 0.7% was polypropylene Homopolymer	Present study
	Pakistan	(PVC) with 51.2%, low-density polyethylene (LDPE) (30.7%), polystyrene (PS) (13.6%), polypropylene homopolymer (PPH) (4.5%)	[12]
	Central Florida, USA	Polyethylene terephthalate (16%), ethylene-co-polypropylene (11%), and cellulose were shown to be more prevalent than the previous two types of polymers, making up 37% of the total particles	[26]
	Antarctic regions (Bird Island, South Georgia and Signy Island, South Orkney Islands)	Discovered several polymers, including polypropylene, polyethylene, polyacrylonitrile, and polyacrylate	[47]
	China	Polyethylene terephthalate (51%), epoxy resin (19%), polyethylene (12%), and alkyd resin (8%)	[48]
	Norway	Polypropylene, polystyrene, and polyethylene variety; polyethylene was identified as the most prevalent form of polymer	[52]

## 4. Conclusions

To our knowledge, this is the first study of MPs in the crops and gizzards of wild birds. The current study found MPs in all samples of crops and gizzards as well in river surface water. Overall, relatively higher numbers of MPs were recovered from gizzards than crops. Four different types and shapes of MP particles were recovered, and fragments were the dominant type in crops and gizzards, while in surface water, fibers were the most abundant type of MPs. In terms of the size range, relatively larger particles (300–500 µm) were abundant in crops, while the dominant size ranges in gizzards and water were 150–300 µm and 50–150 µm, respectively. Duck is a suitable species to assess aquatic plastic pollution. The current study could be a valuable bridge between already existing literature and future recommendations in this regard. As the issue of microplastics and their impact on the environment becomes more apparent, there is an urgent need for further research to address this problem effectively. This study provides new and important information on MP contamination in wild birds. Further research should be conducted to quantitively assess MP contamination in wild birds and to investigate the health implications associated with MP inhalation, particularly because MP pollution is expected to become more severe in the future on a global scale. Future research should include standardized methods, impact on human health, microplastic removal techniques, geographical variations, ecological factors, and other approaches to effectively address this crucial issue.

## Figures and Tables

**Table 1:** Correlation between MP concentration in river water and the crops and gizzards of the ducks.

Correlations				
		Crops	Gizzards	Water
Water	Pearson Correlation	0.242	0.058	1
	Sig.	0.303	0.809	
	N	20	20	20

## Data Availability

All data generated or analyzed during this study were included in this published article.

## References

[1] Arowojolu I.M., de Oliveira Alves I., Benson N.U., Sodré F.F. (2023). Microplastics in aquatic environments: A growing, unresolved concern. Chem. Total Environ..

[2] Ferreira H.C., Lôbo-Hajdu G. (2023). Microplastics in coastal and oceanic surface waters and their role as carriers of pollutants of emerging concern in marine organisms. Mar. Environ. Res..

[3] Bilal M., Qadir A., Yaqub A., Hassan H.U., Irfan M., Aslam M. (2022). Microplastics in water, sediments, and fish at Alpine River, originating from the Hindu Kush Mountain, Pakistan: Implications for conservation. Environ. Sci. Pollut. Res..

[4] de Oliveira C.R.S., da Silva Júnior A.H., Mulinari J., Ferreira A.J.S., da Silva A. (2023). Fibrous microplastics released from textiles: Occurrence, fate, and remediation strategies. J. Contam. Hydrol..

[5] Zitouni N., Cappello T., Missawi O., Boughattas I., De Marco G., Belbekhouche S., Mokni M., Alphonse V., Guerbej H., Bousserrhine N. (2022). Metabolomic disorders unveil hepatotoxicity of environmental microplastics in wild fish Serranus scriba (Linnaeus 1758). Sci. Total Environ..

[6] Yin L., Du L., Wen X., Huang D., Xiao R., Wang Z., Su H., Huang J., Wang G., Tao J. (2023). Occurrence and Effects of Microplastics in Lake Ecosystems: Particular Focus on Migration in Water and Food Chains. Rev. Environ. Cont. Toxicol..

[7] Bilal M., Ul Hassan H., Siddique M.A.M., Khan W., Gabol K., Ullah I., Sultana S., Abdali U., Mahboob S., Khan M.S. (2023). Microplastics in the Surface Water and Gastrointestinal Tract of Salmo trutta from the Mahodand Lake, Kalam Swat in Pakistan. Toxics.

[8] Hanslik L. (2020). Microplastics in Limnic Ecosystems-Investigation of Biological Fate and Effects of Microplastic Particles and Associated Contaminants in Zebrafish (Danio rerio). Ph.D. Thesis.

[9] Irfan M., Qadir A., Mumtaz M., Ahmad S.R. (2020). An unintended challenge of microplastic pollution in the urban surface water system of Lahore, Pakistan. Environ. Sci. Pollut. Res..

[10] Bilal M., Ul Hassan H., Taj M., Rafiq N., Nabi G., Ali A., Gabol K., Shah M.I.A., Ghaffar R.A., Sohail M. (2023). Biological Magnification of Microplastics: A Look at the Induced Reproductive Toxicity from Simple Invertebrates to Complex Vertebrates. Water.

[11] Dellisanti W., Leung M.M., Lam K.W., Wang Y., Hu M., Lo H.S., Fang J.K. (2023). A short review on the recent method development for extraction and identification of microplastics in mussels and fish, two major groups of seafood. Mar. Pollut. Bull..

[12] Bilal M., Taj M., Ul Hassan H., Yaqub A., Shah M.I.A., Sohail M., Rafiq N., Atique U., Abbas M., Sultana S. (2023). First Report on Microplastics Quantification in Poultry Chicken and Potential Human Health Risks in Pakistan. Toxics.

[13] Barboza L.G.A., Lopes C., Oliveira P., Bessa F., Otero V., Henriques B., Raimundo J., Caetano M., Vale C., Guilhermino L. (2020). Microplastics in Wild Fish from North East Atlantic Ocean and Its Potential for Causing Neurotoxic Effects, Lipid Oxidative Damage, and Human Health Risks Associated with Ingestion Exposure. Sci. Total Environ..

[14] Bange A., Backes A., Garthe S., Schwemmer P. (2023). Prey choice and ingestion of microplastics by common shelducks and common eiders in the Wadden Sea World Heritage Site. Mar. Biol..

[15] Cole M., Lindeque P., Fileman E., Halsband C., Galloway T.S. (2015). The impact of polystyrene microplastics on feeding, function and fecundity in the marine copepod *Calanus helgolandicus*. Environ. Sci. Technol..

[16] Khan M.L., Hassan H.U., Khan F.U., Ghaffar R.A., Rafiq N., Bilal M., Khooharo A.R., Ullah S., Jafari H., Nadeem K. (2023). Effects of microplastics in freshwater fishes health and the implications for human health. Braz. J. Biol..

[17] Bilal M., Ali H., Ullah R., Hussain I., Khan B.A. (2021). Overview of Microplastics Threat in Aquatic Animals Since 1960 to 2020. Int. J. Res. Anal. Rev..

[18] Porcino N., Bottari T., Mancuso M. (2022). Is wild marine biota affected by microplastics?. Animals.

[19] Neves D., Sobral P., Ferreira J.L., Pereira T. (2015). Ingestion of microplastics by commercial fish off the Portuguese coast. Mar. Pollut. Bull..

[20] Provencher J.F., Vermaire J.C., Avery-Gomm S., Braune B.M., Mallory M.L. (2018). Garbage in guano? Microplastic debris found in faecal precursors of seabirds known to ingest plastics. Sci. Total Environ..

[21] Ziccardi L.M., Edgington A., Hentz K., Kulacki K.J., Kane Driscoll S. (2016). Microplastics as vectors for bioaccumulation of hydrophobic organic chemicals in the marine environment: A state-of-the-science review. Environ. Toxicol. Chem..

[22] Rummel C.D., Escher B.I., Sandblom O., Plassmann M.M., Arp H.P.H., MacLeod M., Jahnke A. (2019). Effects of leachates from UV-weathered microplastic in cell-based bioassays. Environ. Sci. Technol..

[23] Bour A., Avio C.G., Gorbi S., Regoli F., Hylland K. (2018). Presence of microplastics in benthic and epibenthic organisms: Influence of habitat, feeding mode and trophic level. Environ. Pollut..

[24] Bond A.L. (2016). Diet changes in breeding herring gulls (*Larus argentatus*) in Witless Bay, Newfoundland and Labrador, Canada, over 40 years. Waterbirds.

[25] Masiá P., Ardura A., Garcia-Vazquez E. (2019). Microplastics in special protected areas for migratory birds in the Bay of Biscay. Mar. Pollut. Bull..

[26] Carlin J., Craig C., Little S., Donnelly M., Fox D., Zhai L., Walters L. (2020). Microplastic accumulation in the gastrointestinal tracts in birds of prey in central Florida, USA. Environ. Pollut..

[27] Wang L., Nabi G., Yin L., Wang Y., Li S., Hao Z., Li D. (2021). Birds and plastic pollution: Recent advances. Avian Res..

[28] Susanti R., Yuniastuti A., Fibriana F. (2021). The Evidence of microplastic contamination in Central Javanese local ducks from intensive animal husbandry. Water Air Soil Pollut..

[29] Bustamante T. (2021). Assessing the Presence and Concentration of Microplastics in the Gizzards of Virginia Waterfowl. Ph.D. Thesis.

[30] Faure F., Corbaz M., Baecher H., de Alencastro L.F. (2012). Pollution due to plastics and microplastics in Lake Geneva and in the Mediterranean Sea. Arch. Des. Sci..

[31] D’Souza J.M., Windsor F.M., Santillo D., Ormerod S.J. (2020). Food web transfer of plastics to an apex riverine predator. Glob. Chang. Biol..

[32] Holland E., Mallory M., Shutler D. (2016). Plastics and other anthropogenic debris in freshwater birds from Canada. Sci. Total Environ..

[33] Brookson C.B., de Solla S.R., Fernie K.J., Cepeda M., Rochmana C.M. (2019). Microplastics in the diet of nestling double-crested cormorants (*Phalacrocorax auritus*), an obligate piscivore in a freshwater ecosystem. Can. J. Fish. Aquat. Sci..

[34] Kühn S., Rebolledo E.L.B., van Franeker J.A. (2015). Deleterious effects of litter on marine life. Marine Anthropogenic Litter.

[35] Bond A.L., Provencher J.F., Daoust P.-Y., Lucas Z.N. (2014). Plastic ingestion by fulmars and shearwaters at Sable Island, Nova Scotia, Canada. Mar. Pollut. Bull..

[36] Codina-García M., Militão T., Moreno J., González-Solís J. (2013). Plastic debris in Mediterranean seabirds. Mar. Pollut. Bull..

[37] Herzke D., Anker-Nilssen T., Nøst T.H., Götsch A., Christensen-Dalsgaard S., Langset M., Fangel K., Koelmans A.A. (2016). Negligible Impact of Ingested Microplastics on Tissue Concentrations of Persistent Organic Pollutants in Northern Fulmars off Coastal Norway. Environ. Sci. Technol..

[38] Tanaka K., Takada H., Yamashita R., Mizukawa K., Fukuwaka M.-A., Watanuki Y. (2013). Accumulation of plastic-derived chemicals in tissues of seabirds ingesting marine plastics. Mar. Pollut. Bull..

[39] Lindborg V.A., Ledbetter J.F., Walat J.M., Moffett C. (2012). Plastic consumption and diet of Glaucous-winged Gulls (*Larus glaucescens*). Mar. Pollut. Bull..

[40] Kühn S., van Franeker J.A. (2012). Plastic ingestion by the northern fulmar (*Fulmarus glacialis*) in Iceland. Mar. Pollut. Bull..

[41] Lusher A. (2015). Microplastics in the marine environment: Distribution, interactions and effects. Marine Anthropogenic Litter.

[42] Reynolds C., Ryan P.G. (2018). Micro-plastic ingestion by waterbirds from contaminated wetlands in South Africa. Mar. Pollut. Bull..

[43] Collard F., Bangjord G., Herzke D., Gabrielsen G.W. (2022). Plastic burdens in northern fulmars from Svalbard: Looking back 25 years. Mar. Pollut. Bull..

[44] Tokunaga Y., Okochi H., Tani Y., Niida Y., Tachibana T., Saigawa K., Katayama K., Moriguchi S., Kato T., Hayama S.-I. (2022). Airborne Microplastics Detected in the Lungs of Wild Birds in Japan. Chemosphere.

[45] Zhao S., Zhu L., Li D. (2016). Microscopic anthropogenic litter in terrestrial birds from Shanghai, China: Not only plastics but also natural fibers. Sci. Total Environ..

[46] Deoniziak K., Cichowska A., Nied’zwiecki S., Pol W. (2022). Thrushes (Aves: Passeriformes) as indicators of microplastic pollution interrestrial environments. Sci. Total Environ..

[47] Bessa F., Ratcliffe N., Otero V., Sobral P., Marques J.C., Waluda C.M., Trathan P.N., Xavier J.C. (2019). Microplastics in gentoo penguins from the Antarctic region. Sci. Rep..

[48] Liu S.L., Jian M.F., Zhou L.Y., Li W.H., Wu X.E., Rao D. (2019). Pollution characteristics of microplastics in migratory bird habitats located within Poyang Lake wetlands. Huan Jing Ke Xue Huanjing Kexue.

[49] Zhu C., Li D., Sun Y., Zheng X., Peng X., Zheng K., Hu B., Luo X., Mai B. (2019). Plastic debris in marine birds from an island located in the South China Sea. Mar. Pollut. Bull..

[50] Susanti N.K.Y., Mardiastuti A., Hariyadi S. (2022). Microplastics in Digestive System of Little-black cormorant (*Phalacrocorax sulcirostris*) in Pulau Rambut Sanctuary. IOP Conference Series: Earth and Environmental Science.

[51] Zhao S., Wang T., Zhu L., Xu P., Wang X., Gao L., Li D. (2019). Analysis of suspended microplastics in the Changjiang Estuary:Implications for riverine plastic load to the ocean. Water Res..

[52] Collard F., Husum K., Eppe G., Malherbe C., Hallanger I.G., Divine D.V., Gabrielsen G.W. (2021). Anthropogenic particles in sediment from an Arctic fjord. Sci. Total Environ..

[53] Allahvaisi S., Dogan F. (2012). Polypropylene in the industry of food packaging. Polypropylene.

